# BH3 profiling discriminates the anti-apoptotic status of 5-fluorouracil-resistant colon cancer cells

**DOI:** 10.3892/or.2019.7373

**Published:** 2019-10-15

**Authors:** Kazuma Ishikawa, Yutaka Kawano, Yohei Arihara, Tomohiro Kubo, Kohichi Takada, Kazuyuki Murase, Koji Miyanishi, Masayoshi Kobune, Junji Kato

**Affiliations:** 1 Department of Medical Oncology, Sapporo Medical University School of Medicine, Sapporo, Hokkaido 060-8543, Japan; 2 Department of Internal Medicine, Health Sciences University of Hokkaido, Sapporo, Hokkaido 002-8072, Japan; 3 Department of Hematology, Sapporo Medical University School of Medicine, Sapporo, Hokkaido 060-8543, Japan

**Keywords:** BH3 profiling, BCLXL, colon cancer, 5-fluorouracil, drug resistance

## Abstract

5-Fluorouracil (5-FU) is a cytotoxic anticancer drug commonly used for patients with advanced colon cancer. This drug effectively reduces the size of tumors to a certain degree; however, cancer cells can gradually acquire resistance, resulting in disease progression. To identify the mechanism of 5-FU resistance, we established three 5-FU-resistant colon cancer cell lines and analyzed both apoptosis-related protein expression levels and BH3 profiling. These 5-FU-resistant colon cancer cell lines acquired apoptotic resistance to 5-FU. Although apoptosis-related protein expression levels were altered in each 5-FU-resistant colon cancer cell line variably, BH3 profiling indicated BCLXL dependence in 5-FU-resistant HT-29 cells only. Functional BCLXL inhibition in 5-FU-resistant HT-29 cells not only sensitized the cells to apoptosis but also overcame 5-FU resistance. The apoptotic BIM protein was preferentially sequestered, thereby resulting in acquired dependence on BCLXL for survival. Additionally, *in vivo* models showed that BCLXL inhibition controlled tumor progression. These results indicate that BH3 profiling facilitates the identification of the functional role of anti-apoptotic proteins during drug resistance and has clinical implications for colon cancer in targeting specific proteins such as BCLXL.

## Introduction

Colon cancer is the third most commonly diagnosed cancer in the world (1) and the second most commonly diagnosed cancer in Japan (2). The prognosis of advanced colon cancer with metastasis remains poor, with the overall 5-year survival rate being only 18.8% (3). Standard treatment of unresectable advanced or recurrent colon cancer is systemic chemotherapy. The recent development of several chemotherapy regimens such as FOLFIRI and FOLFOX, which are combined with bevacizumab, cetuximab, or panitumumab, has clearly prolonged patient survival (3). In Japan, fourth or fifth line continuous chemotherapy regimens are administered for unresectable advanced colon cancer such that all usable anticancer drugs are administered. Among them, 5-fluorouracil (5-FU) is a core cytotoxic drug that is included in all first line regimens. 5-FU is an analogue of uracil, which is one of the four bases found in RNA; it is also utilized more in tumors than in normal tissue (4). After entering the cell, 5-FU is converted to several metabolites (5), which leads to misincorporation into RNA and DNA, thereby inhibiting thymidine synthase and initiating apoptosis. In general, cytotoxic anticancer drugs are effective with respect to killing cancer cells at the beginning of treatment. However, tumors gradually fail to respond to these drugs after drug resistance sets in. Therefore, understanding the mechanism of resistance to anticancer drugs is indispensable for uncovering more efficacious treatments for refractory cancer.

In the present study, we established three colon cancer cell lines with acquired resistance to continuous 5-FU treatment and analyzed the mechanism of 5-FU resistance in terms of anti-apoptosis using a well-designed functional apoptotic assay-BH3 profiling.

## Materials and methods

### 

#### Cell lines

Colorectal adenocarcinoma cell lines DLD-1 (ATCC CCL-221TM), HCT-15 (ATCC CCL-225TM) and HT-29 (ATCC HTB-38TM) were obtained from the American Type Culture Collection and cultured at 37°C in a humidified 5% CO_2_ incubator in either RPMI (DLD-1, HCT-15) or DMEM (HT-29) medium supplemented with 10% FBS, 10 mM L-glutamine, 100 U/ml penicillin, and 100 µg/ml streptomycin (all media and supplements from Sigma-Aldrich; Merck KGaA).

#### Generation of the 5-FU-resistant cell line

The 5-FU-resistant colon cancer cell line was developed as previously reported (6). Briefly, parental colon cancer cell lines were treated with gradually increasing concentrations of 5-FU (Sigma-Aldrich; Merck KGaA). The initial 5-FU concentration added to cells was 10% of the IC_50_; this concentration was gradually increased. Colon cancer cell lines resistant to 16 µM of continuous 5-FU administered continuously were established according to a previous study (7).

#### Cell viability assay

Each colon cancer cell line was treated for 72 h with 5-FU at the indicated concentrations in 96-well plates (Thermo Fisher Scientific, Inc.). Subsequently, cell proliferation was determined using the Premix WST-1 Cell Proliferation Assay (Takara Bio Inc., Kusatsu, Shiga, Japan) and an Infinite M1000 PRO microplate reader (Tecan Japan, Kawasaki, Kanagawa, Japan). The half-maximal inhibitory concentration (IC_50_) of 5-FU was defined as the drug concentration resulting in 50% cell survival relative to that of untreated cells. Triplicate wells were treated with various drug concentrations and average IC_50_ values were determined. As known antagonists of BCL2 and BCLXL in *in vitro* studies, ABT-199 (Selleck Chemicals) and WEHI-539 hydrochloride (MedChem Express) were used and the IC_50_ values of each drug were obtained, respectively.

#### Apoptosis assays

Parental and 5-FU-resistant colon cancer cells were allowed to adhere to 6-well plates for 24 h and cells were treated with either 5-FU or WEHI-539 hydrochloride as indicated. Cells were then stained with a phycoerythrin-conjugated Annexin V antibody and 7-AAD (BD Pharmingen; BD Biosciences). Apoptotic cells were analyzed using a BD FACSCanto II flow cytometer (BD Biosciences) with FACSDiva software (BD Biosciences). The percentage of apoptotic cells was calculated by dividing the percentage of either Annexin V-positive or 7-AAD positive cells by the total cells. Apoptosis was also assessed using the Caspase-Glo^®^ 3/7 Assay (Promega). Five thousand cells were plated in white-walled 96-well round plates (Thermo Fisher Scientific, Inc.) and treated with the drugs as indicated. After incubation, 100 µl of Caspase-Glo^®^ reagent was added to each well and the contents of the well were gently mixed with a plate shaker at 50 × g for 30 sec; this was followed by incubation at 20°C room temperature for 1 h. The luminescence of each sample was measured using an Infinite M1000 PRO microplate reader. The caspase inhibitor Q-VD-OPH (Bay Bioscience, Kobe, Hyogo, Japan) was also used.

#### Western blot analysis

Western blotting was performed as previously described (8). Briefly, separated proteins were transferred to polyvinylidene difluoride membranes and blotted with specific antibodies to detect BCL2 (at dilution of 1:500; Thermo Fisher Scientific, Inc.; #13-8800), BCLW (1:1,000; Cell Signaling Technology; cat. no. 2724), BCLXL (1:1,000; Cell Signaling Technology; cat. no. 2764), MCL1 (1:1,000; Cell Signaling Technology; cat. no. 5453), BAK (1:1,000; Cell Signaling Technology; cat. no. 12105), BAX (1:1,000; Cell Signaling Technology; cat. no. 5023), BIM (1:1,000; Cell Signaling Technology; cat. no. 2933), BID (1:1,000; Cell Signaling Technology; #2002), BAD (1:1,000; Cell Signaling Technology; cat. no. 9292), NOXA (1:1,000; Cell Signaling Technology; cat. no. 14766), PUMA (1:1,000; Cell Signaling Technology; cat. no. 12450), BMF [1:1,000; Abcam; cat. no. EPR10930 (2)], HRK (1:200; R&D Systems; cat. no. AF851), and actin (1:3,000; Santa Cruz Biotechnology; cat. no. sc1615). After incubation with either horseradish peroxidase-linked anti-rabbit IgG (1:2,000; Cell Signaling Technology; cat. no. 7074S) or anti-mouse IgG (1:2,000; Cell Signaling Technology; cat. no. 7076S), the membranes were stained with ECL Select Western Blotting Detection Reagent (GE Healthcare UK Ltd.). Finally, the bands were imaged either by exposing membranes to BIOMAX XAR film (Sigma-Aldrich; Merck KGaA) and developing the images using a Kodak X-OMAT 1000 Processor (Kodak via Thermo Fisher Scientific, Inc.) or using an LAS-4000UV mini (GE Healthcare UK Ltd.) and MultiGauge software (Fujifilm, Tokyo, Japan).

#### BCL2-homology domain 3 (BH3) profiling

We conducted fluorescence activated cell sorting (FACS)-based BH3 profiling as previously described (9,10). Nine BH3 peptides were obtained as HPLC-purified products from Sigma-Aldrich; Merck KGaA (Table I). All peptides were dissolved in dimethyl sulfoxide (DMSO; Sigma-Aldrich; Merck KGaA) as 1 mM stock solutions and stored at −80°C. As a control for mitochondrial depolarization, p-trifluoromethoxy carbonyl cyanide phenyl hydrazine (FCCP) was used. Two hundred thousand parental and 5-FU-resistant colon cancer cells were suspended in TE-B buffer [300 mM trehalose, 10 mM HEPES-KOH (pH 7.7), 80 mM KCl, 1 mM EDTA, 1 mM EGTA, 0.1% bovine serum albumin (BSA), and 5 mM succinate; all from Sigma-Aldrich; Merck KGaA] containing 0.001% digitonin (Sigma-Aldrich; Merck KGaA) and 20 µg/ml oligomycin (Sigma-Aldrich; Merck KGaA), followed by incubation with each BH3 peptide at a final concentration of 10 µM for 30 min. After staining the cells with 25 nM tetramethylrhodamine ethyl ester (Invitrogen/Thermo Fisher Scientific, Inc.), fluorescence intensities were analyzed using a BD FACSCanto II flow cytometer (BD Biosciences) with FACSDiva software (BD Biosciences). The percentage of relative mitochondrial depolarization was calculated using the following equation:

$$\%\;\text{mitochondrial}\;\text{depolarization}=\;\frac{100\times (\text{DMSO}\;(\text{fv})\;-\;X(\text{fv}))}{\left(\text{DMSO}\;(\text{fv})-\text{FCCP}\;(\text{fv})\right)}$$

where DMSO(fv) is the mean fluorescence value as the negative control, X(fv) indicates the fv as the tested BH3 peptide, and FCCP(fv) indicates the fv as the positive control.

#### Inhibition of BCLXL expression via small-interfering RNA (siRNA) transfection

One million parental and 5-FU-resistant HT-29 cells in 6-well plates were transfected with either siRNAs targeting human BCLXL (Dharmacon; siBCLXL#1: D-003458-03, siBCLXL#2: D-003458-30) or a non-targeting siRNA (Dharmacon; siNT: D-001210-03-05) using RNAi Max reagent (Invitrogen; Thermo Fisher Scientific, Inc.) according to the manufacturer's instructions.

#### Immunoprecipitation

BCLXL immunoprecipitation experiments were conducted using the ImmunoCruz™ IP/WB Optima F System (Santa Cruz Biotechnology; #SC-45043). Separated total proteins (1,000 µg) were incubated with a complex of BCLXL antibody and IP matrix overnight at 4°C. After incubation and centrifugation, the eluted supernatant was subjected to western blotting for BID, BIM, and BCLXL.

#### Tumor xenograft study

Fifteen female BALB/c nu/nu mice (six weeks of age, 18–20 g/each) were purchased from Sankyo Labo Service Corporation, Inc. (Tokyo, Japan). Five million 5-FU-resistant HT-29 cells suspended in 100 µl DMEM and 100 µl Matrigel (BD Biosciences) were injected subcutaneously into the left flank. In place of WEHI-539, which has a labile and potentially toxic hydrazone moiety, we used another BCLXL inhibitor, A-1155463, which is known to be efficacious on tumor growth suppression *in vivo* (11). Mice were divided into three experimental groups: Control (vehicle-treated), 5-FU, and A-1155463. When tumor volumes reached 100 mm^3^, administration of either intravenous 5-FU (50 mg/kg) injections once a week or intraperitoneal A-1155463 (5 mg/kg) injections every day was conducted for four weeks as previously reported (11,12). Tumor size and body weight were measured throughout the experimental period. Tumor volumes were calculated with the following formula:

$$\text{Tumor}\;\text{volume}\;({\text{mm}}^{3})=(\text{Length}\;(\text{mm})x\;\text{Width}\;(\text{mm})\;x\;\text{Height}\;(\text{mm})\;\times \;\pi )/6$$

Percent volume was calculated with the following formula:

$$\%\text{volume}\;=\;100\;x\;(\text{Tumor}\;\text{volume}\;({\text{mm}}^{3})\;\text{of}\;\text{day})/(\text{Tumor}\;\text{volume}\;({\text{mm}}^{3})\;\text{of}\;\text{day}\;0)$$

Mice were sacrificed when the tumor diameter reached 20 mm or when the study period finished. Tumors and other organs including the lung and liver were isolated, followed by hematoxylin and eosin staining. Apoptotic cells were analyzed by TUNEL staining using an in situ apoptosis detection kit (Takara Bio Inc.). The percentage of TUNEL-positive cells was calculated by counting cells in 5 different fields. The animal study was conducted according to protocols approved by the Animal Ethics Committee of the Sapporo Medical University School of Medicine (Protocol no. 17-142). Euthanasia was conducted by high concentration carbon dioxide and all efforts were made to minimize suffering.

#### Statistical analysis

All data represent the mean ± SD. For comparisons between two groups, two-tailed unpaired Student's t-tests were performed. For multiple comparisons of *in vitro* experiments, one way analysis of variance followed by Tukey test was conducted. IC_50_ values for each drug tested and dose-response curves were analyzed using Graph-Pad PRISM 5 (GraphPad Software, Inc., La Jolla, CA, USA). For *in vivo* experiments, one way analysis of variance followed by Dunnett's post hoc test was adopted for comparison with the control group. All statistical analyses were performed using SPSS software version 21 (IBM, Armonk, NY, USA).

## Results

### 

#### Enhanced survival in three 5-FU-resistant colon cancer cell lines

**Figure 1.**
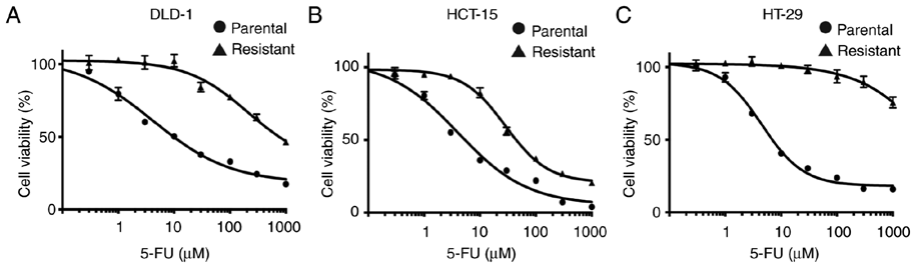
Establishment of three 5-FU-resistant colon cancer cell lines. Both parental and 5-FU-resistant DLD-1 (A), HCT-15 (B) and HT-29 (C) cells were treated with different concentrations of 5-FU for 72 h, followed by measurement of cell viability. Each point indicates the mean ± SD (n=3). 5-FU, 5-fluorouracil.

We established three colon cancer cell lines which were resistant to 16 µM continuous 5-FU treatment. The 5-FU IC_50_ values of resistant colon cancer cells were markedly higher than those of the parental cells (DLD-1: 5-FU-resistant 776.0 µM, parental 10.3 µM; HCT-15: 5-FU-resistant 44.2 µM, parental 4.1 µM; HT-29: 5-FU-resistant >1,000 µM, parental 7.2 µM, respectively) (Fig. 1A-C).

#### Resistance to apoptosis in 5-FU-resistant colon cancer cells and BCL2 protein induction in 5-FU-resistant HT-29 cells

**Figure 2.**
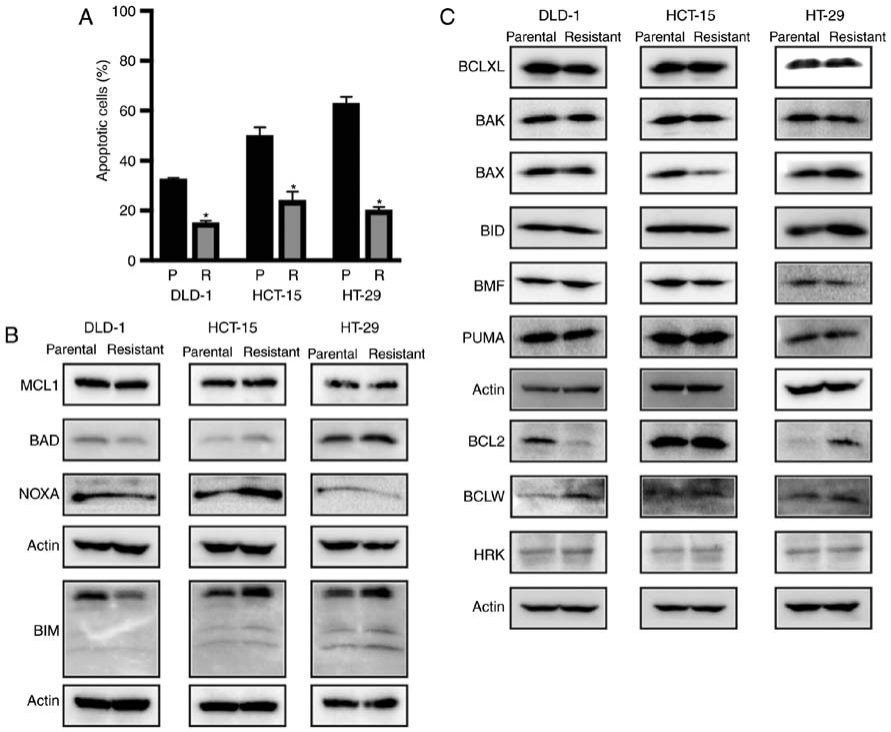
5-Fluorouracil (5-FU)-resistant colon cancer cells acquire resistance to apoptosis. (A) Parental (P; black) and 5-FU-resistant (R; gray) colon cancer cell lines were treated with 100 µM 5-FU for 72 h, followed by assessment of apoptosis by flow cytometry. All data represent the mean ± SD (n=3). *P<0.01, a significant difference when compared to parental colon cancer cells. (B and C) Western blot analysis of anti-apoptosis- and apoptosis-related protein expression in three colon cancer cell lines of both parental and 5-FU-resistant cells. Each actin band image was used as a loading control for the band image of the proteins of interest.

To evaluate the mechanisms of 5-FU resistance, we first verified the occurrence of apoptosis in parental cells and three 5-FU-resistant colon cancer cell lines. Each 5-FU-resistant colon cancer cell line contained significantly fewer Annexin-positive apoptotic cells compared with the parental cells, suggesting that apoptotic resistance had been acquired by 5-FU-resistant colon cancer cell lines (Figs. 2A and S1). We then examined expression alterations in both anti-apoptotic and apoptotic proteins between parental and 5-FU-resistant colon cancer cells (Fig. 2B and C). Western blot analysis showed that BCLXL and MCL1 protein expression levels were similar between the parental and 5-FU-resistant cells in the three colon cancer cell lines. Whereas BCL2 protein expression in 5-FU-resistant DLD-1 cells was decreased, both BCLW expression in DLD-1 and BCL2 protein expression in HT-29 was increased in the 5-FU-resistant cells. The expression of apoptosis-related proteins, e.g., BAX was decreased whereas BIM, BAD, and NOXA expression was increased in the 5-FU-resistant HCT-15 cells. Whereas NOXA expression was decreased in the 5-FU-resistant HT-29 cells, BIM expression was increased, suggesting that the expression patterns of both anti-apoptotic and apoptotic proteins were altered during the acquisition of 5-FU resistance in the three colon cancer cell lines. From this analysis of apoptosis-related protein expression, it may be difficult to identify specific apoptotic responses to 5-FU resistance solely by quantifying protein expression.

#### BH3 profiling reveals that 5-FU-resistant HT-29 cells are dependent on BCLXL for survival

**Figure 3.**
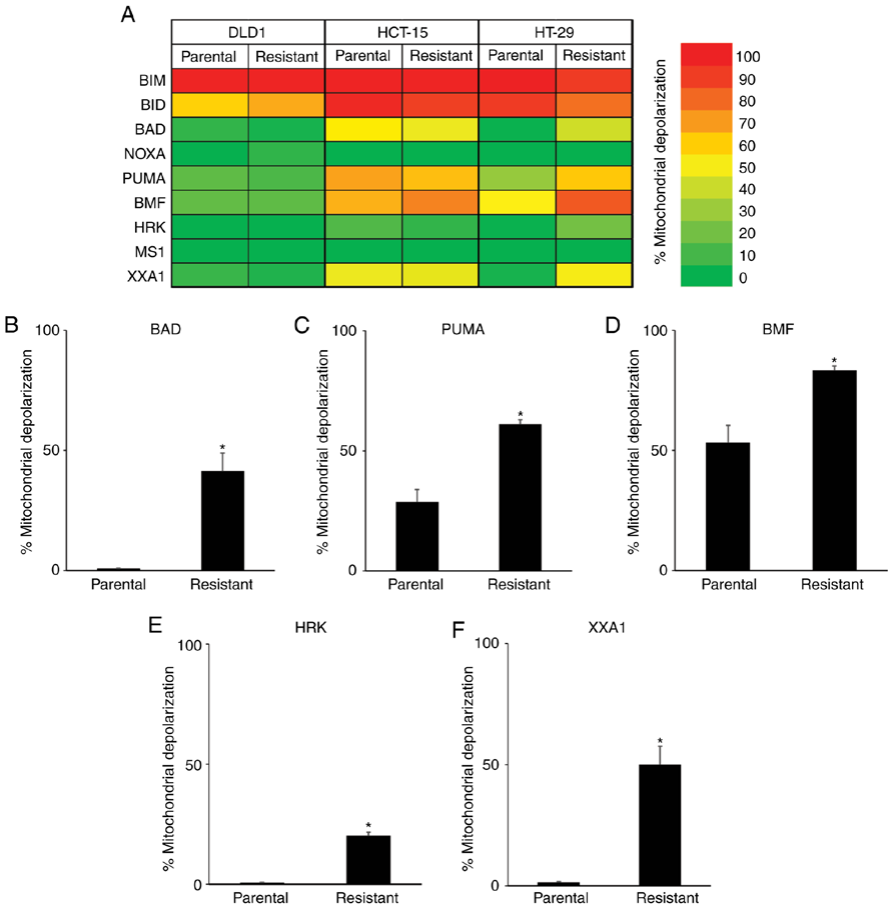
BH3 profiling determines that mitochondrial priming is dependent on BCLXL in 5-fluorouracil (5-FU)-resistant HT-29 cells. (A) Heat map for mitochondrial responses to nine BH3 peptides in three colon cancer cell lines of both parental and 5-FU-resistant cells. Bar graph representing the percentage of mitochondrial depolarization in parental and 5-FU-resistant HT-29 cells when treated with BAD (B), PUMA (C), BMF (D), HRK (E) and XXA1 (F). All data represent the mean ± SD (n=3). *P<0.01, a significant difference when compared to parental HT-29 cells.

To further clarify the relationship between 5-FU resistance and apoptosis-related proteins, we conducted a functional analysis via BH3 profiling (Fig. 3A). When treated with BIM and BID BH3 peptides, which interact directly with BAK and BAX proteins resulting in the release of cytochrome *c* into the cytoplasm, strong mitochondrial depolarization was observed in the parental and all 5-FU-resistant cells. We then treated cells with BAD, NOXA, PUMA, BMF, and HRK BH3 peptides, which have variable affinities for anti-apoptotic proteins, and compared the mitochondrial depolarization between parental and 5-FU-resistant colon cancer cells. In DLD-1 and HCT-15 cells, no significant differences were observed between parental and 5-FU-resistant cells when treated with these BH3 peptides. However, greater mitochondrial depolarization was observed in 5-FU-resistant HT-29 cells when treated with BAD (Fig. 3B), PUMA (Fig. 3C), and BMF (Fig. 3D) BH3 peptides, which was correlated with dependency on BCL2, BCLXL or BCLW. Furthermore, treatment with HRK peptide, which binds specifically to BCLXL, caused a small increase in mitochondrial depolarization in the 5-FU-resistant HT-29 cells (Fig. 3E mitochondrial depolarization: Parental, 0.7%; 5-FU-resistant, 20.3%). To further clarify the dependency on BCLXL protein, HT-29 cells were treated with the XXA1 peptide, which has been structurally identified (13) as a specific inhibitor of BCLXL. Significant differences in mitochondrial depolarization were observed between the parental and 5-FU-resistant HT-29 cells (Fig. 3F mitochondrial depolarization: Parental, 1.5%; 5-FU-resistant, 50.0%). As for DLD-1 and HCT-15, there were no differences in mitochondrial depolarization between parental and 5-FU-resistant cells when treated with XXA1 peptide. We also treated cells with MS1 peptide as a specific MCL inhibitor (14); no changes in mitochondrial depolarization were observed in both parental and 5-FU-resistant colon cancer cells. Taken together, results of the BH3 profiling of 5-FU-resistant HT-29 cells revealed strong dependency on BCLXL for cell survival compared with parental cells, while no relationship to apoptosis-related protein expression profiles was determined, especially BCLXL protein expression. We then focused on the BCLXL dependence of 5-FU-resistant HT-29 colon cancer cells.

#### Inhibition of BCLXL selectively induces apoptosis in 5-FU-resistant HT-29 cells

**Figure 4.**
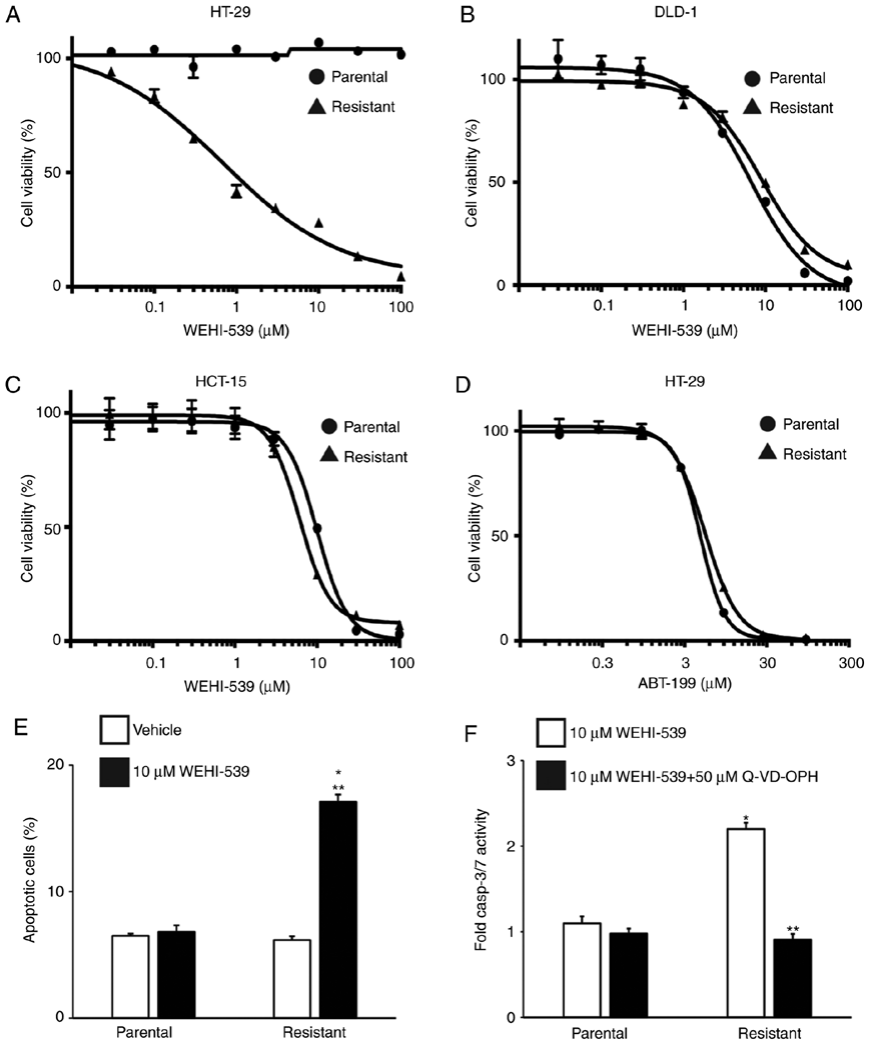
A BCLXL inhibitor not only inhibits proliferation but also promotes apoptosis in 5-FU-resistant HT-29 cells. Both parental and 5-FU-resistant HT-29 (A), DLD-1 (B) and HCT-15 (C) cells were treated with different concentrations of the BCLXL inhibitor, WEHI-539, for 72 h, followed by measurement of cell viability. (D) Parental and 5-FU-resistant HT-29 cells were treated with different concentrations of the BCL2 inhibitor ABT-199 for 72 h, followed by measurement of cell viability. Each point indicates the mean ± SD (n=3). (E) Parental and 5-FU-resistant HT-29 cells were treated with or without 10 µM WEHI-539 for 48 h, followed by the assessment of apoptosis by flow cytometry. *P<0.01, statistical significance when compared to parental HT-29 cells; **P<0.01, statistical significance when compared to vehicle-treated cells. (F) Caspase3/7 activity in parental and 5-FU-resistant HT-29 cells when treated with 10 µM WEHI-539, with or without the caspase inhibitor (50 µM Q-VD-OPH) for 24 h. Values are fold-changes in caspase3/7 activity compared to those with medium treatment only. *P<0.01, statistical significance when compared to parental HT-29 cells; **P<0.01, statistical significance when compared to 5-FU-resistant HT-29 cells without the caspase inhibitor (vehicle). All data represent the mean ± SD (n=3). 5-FU, 5-fluorouracil.

To confirm the BCLXL dependency in 5-FU-resistant HT-29 cells, we first treated parental and 5-FU-resistant colon cancer cells with the BCLXL-specific inhibitor WEHI-539 and assessed the effect of this inhibition on cell survival. As expected, the IC_50_ of WEHI-539 in 5-FU-resistant HT-29 cells was markedly lower compared to that in parental cells (Fig. 4A: 5-FU-resistant, 0.66 µM; parental, >100 µM), whereas no difference in IC_50_ values between parental and 5-FU-resistant cells was observed in DLD-1 (Fig. 4B: 5-FU-resistant, 9.94 µM; parental, 7.12 µM) and HCT-15 (Fig. 4C: 5-FU-resistant, 9.83 µM; parental, 6.31 µM) cells. To rule out any possibility of the contribution of induced BCL2 expression to 5-FU resistance in HT-29 cells as shown in Fig. 2B, we also treated HT-29 cells with the BCL2-specific inhibitor ABT-199 (Fig. 4D). No marked differences in IC_50_ values were observed between the parental and 5-FU-resistant HT-29 cells (IC_50_: Parental, 16.5 µM; 5-FU-resistant, 13.5 µM), suggesting that these inhibitory data support the result of the BH3 profiling rather than that of the anti-apoptotic protein expression profiles. We then examined the effect of WEHI-539 on apoptosis in 5-FU-resistant HT-29 cells to examine how BCLXL inhibition could kill these cells. An significantly increased percentage of apoptotic cells was observed in the 5-FU-resistant HT-29 cells, whereas no additional Annexin V-positive cells were noted in the parental cells (Fig. 4E). Furthermore, the activities of caspase-3 and caspase-7, both of which are key effectors of apoptosis, were upregulated in the 5-FU-resistant HT-29 cells when treated with WEHI-539 and were completely inhibited through co-treatment with the caspase inhibitor Q-VD-OPH (Fig. 4F). These results indicate that BCLXL, but not BCL2, exerts substantial anti-apoptotic functions in 5-FU-resistant HT-29 cells.

#### Restoration of 5-FU sensitivity by inhibiting BCLXL in 5-FU-resistant HT-29 cells

**Figure 5.**
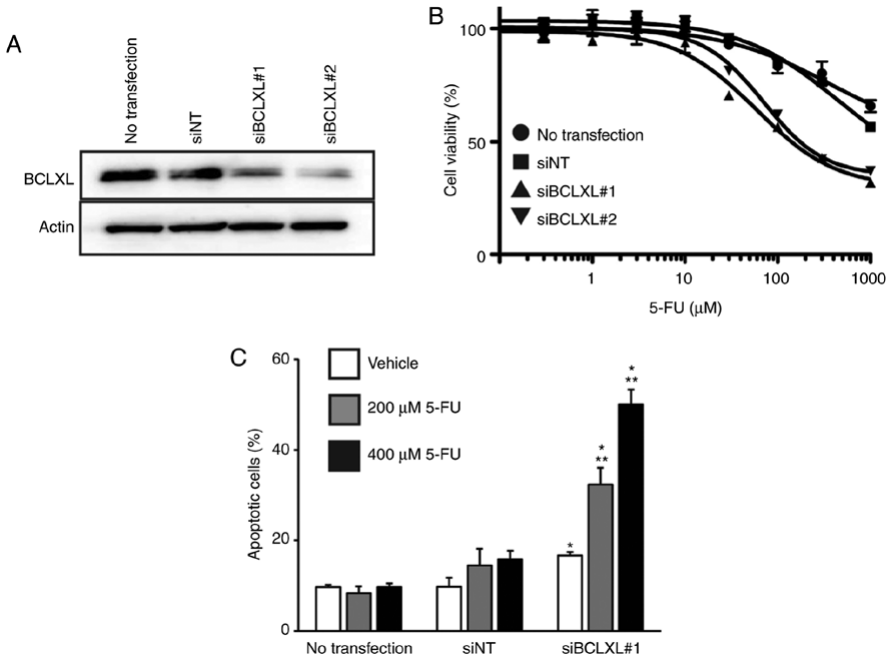
A BCLXL inhibitor but not a BCL2 inhibitor sensitizes 5-FU-resistant HT-29 cells to apoptosis. (A) Western blot analysis of BCLXL siRNA-transfected (siBCLXL#1 and siBCLXL#2) 5-FU-resistant HT-29 cells. (B) siBCLXL transfected 5-FU-resistant HT-29 cells were treated with different concentrations of 5-FU for 72 h, followed by measurements of cell viability. Each point indicates the mean ± SD (n=3). (C) siBCLXL#1 transfected 5-FU-resistant HT-29 cells were treated with either 200 or 400 µM 5-FU for 72 h, followed by the assessment of apoptosis by flow cytometry. *P<0.01, statistical significance when compared to non-targeting siRNA (siNT)-transfected cells; **P<0.01, statistical significance when compared to vehicle-treated 5-FU-resistant HT-29 cells. All data represent the mean ± SD (n=3). 5-FU, 5-fluorouracil.

We then inhibited BCLXL protein expression in 5-FU-resistant HT-29 cells through siRNA transfection (Fig. 5A), followed by treatment with 5-FU. The IC_50_ of 5-FU in the resistant HT-29 cells was markedly decreased following BCLXL inhibition compared to that in both cells transfected with non-targeting siRNA (siNT) and untransfected HT-29 cells (Fig. 5B: siBCLXL#1 transfected, 174.9 µM; siBCLXL#2 transfected, 236.5 µM; siNT transfected, >1,000 µM; untransfected, >1,000 µM). Furthermore, a significantly increased percentage of apoptotic cells was observed following BCLXL inhibition during 5-FU treatment (Fig. 5C), suggesting that BCLXL downregulation restored 5-FU sensitivity in the resistant HT-29 cells by enhancing their vulnerability to apoptosis.

#### Sequestration of BIM by BCLXL mediates sensitization to apoptosis in 5-FU-resistant HT-29 cells

**Figure 6.**
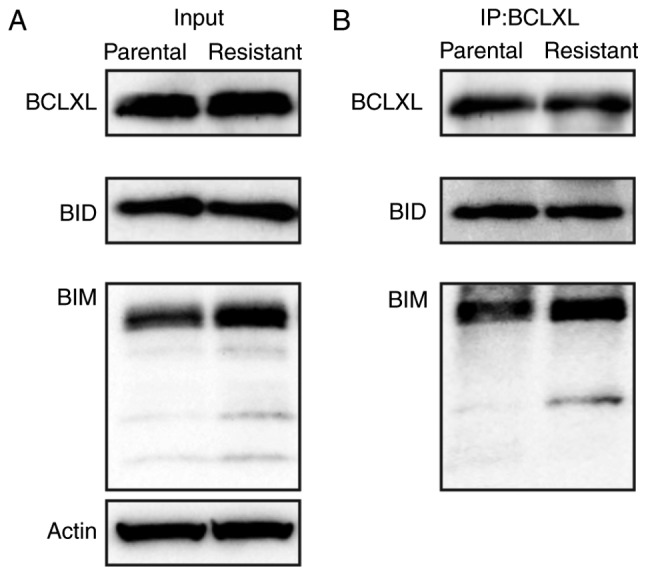
Induced BIM preferentially binds to BCLXL in 5-FU-resistant HT-29 cells. (A) Immunoblot analysis using whole cell lysate (Input) and (B) BCLXL immunoprecipitation analysis of parental and 5-FU-resistant HT-29 cell lines. Actin was used as a loading control for the input analysis shown in A. 5-FU, 5-fluorouracil.

To examine whether an interaction between BCLXL and an apoptotic protein would account for functional BCLXL dependency, we performed immunoprecipitation on cell extracts of parental and 5-FU-resistant HT-29 cells using a BCLXL antibody and examined the binding of apoptotic BIM and BID proteins. As shown in Fig. 6A and B, BIM protein preferentially bound to BCLXL in the 5-FU-resistant HT-29 cells compared to the parental cells. In summary, BCLXL dependence in 5-FU-resistant HT-29 cells was mediated through the sequestration of BIM by BCLXL.

#### Inhibition of BCLXL controls tumor growth in the 5-FU-resistant HT-29 cells

**Figure 7.**
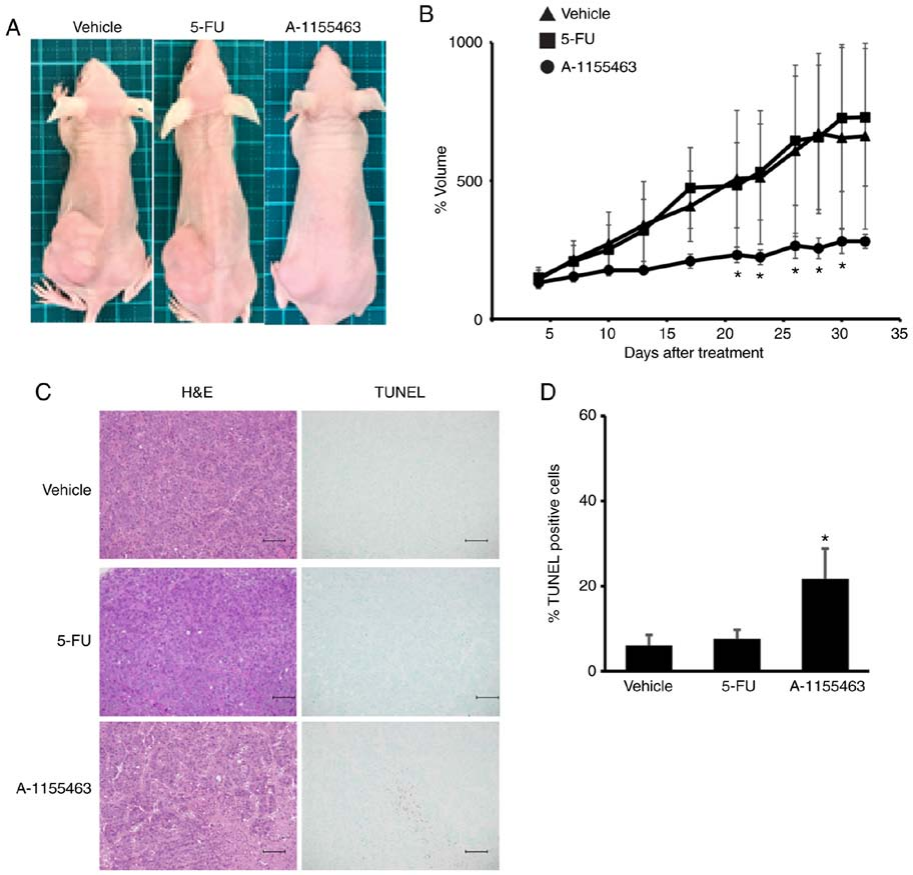
BCLXL inhibitor controls the growth of 5-fluorouracil (5-FU)-resistant HT-29 cells *in vivo*. After engraftment with tumors of 5-FU-resistant HT-29 cells, BALB/c nu/nu mice were divided into three experimental groups; Vehicle (vehicle-treated/control), 5-FU, and A-1155463 groups. (A) Representative images of subcutaneous tumors at day 32 after treatment. (B) Tumor volumes were calculated by measuring the length, height, and width. Data represent the mean ± SD (n=5). *P<0.05, statistical significance when compared to vehicle-treated mice, based on one way analysis of variance followed by Dunnett's post hoc test. (C) Histologic analysis of tumor sections stained with hematoxylin and eosin (H&E) and TUNEL. Scale bars, 100 µm. (D) Statistical analysis of TUNEL staining. *P<0.05, statistical significance when compared to vehicle-treated mice, based on one way analysis of variance followed by Dunnett's post hoc test.

We further tested the effect of BCLXL inhibition on the growth of 5-FU-resistant HT-29 cells using a tumor xenograft model. Prior to our *in vivo* study, we found that the A-1155643 IC_50_ values of 5-FU-resistant HT-29 cells were markedly lower compared to those in parental cells (5-FU-resistant, 1.2 µM; parental, >100 µM). After transplantation of these cells into the left flank, mice were treated for four weeks with either 5-FU or BCLXL inhibitor A-1155643, respectively. Whereas tumor volume gradually increased in the mice treated with 5-FU, similar to vehicle-treated mice, BCLXL inhibitor-treated mice showed stable tumor size as well as a significantly different size compared with vehicle-treated mice (Figs. 7A and B and S2). A significant increase in TUNEL-positive apoptotic tumor cells was also observed in mice treated with A-1155643 (Fig. 7C and D). A-1155643 treatment was well tolerated, without significantly decreasing body weight (day 28 body weight: Vehicle, 17.9±1.8 g; 5-FU treatment, 18.3±1.7 g; A-1155643 treatment, 17.7±1.9 g, respectively; Fig. S3). Also, no severe bleeding in the lung and liver (Fig. S4), which usually occurs due to thrombocytopenia, was observed in the BCLXL inhibitor-treated mice.

## Discussion

In the present study, we presented a new strategy to identify the mechanisms of 5-FU resistance, namely, BH3 profiling. Results of this profiling identified strong dependence on BCLXL regardless of induced BCL2 protein expression in 5-FU-resistant HT-29 cells among three 5-FU-resistant colon cancer cell lines. Furthermore, this BCLXL dependence may facilitate the treatment of 5-FU-resistant colon cancer cells by BCLXL inhibitors *in vitro* and *in vivo*. As reported in a previous study (15), our results suggest that analysis of the expression patterns of apoptosis-related proteins alone is insufficient to explain how cancer cells survive.

In clinical settings, anticancer drugs are continued until they no longer elicit a response by the cancer cells or cause severe adverse effects in the treated patient. During prolonged drug treatment, certain cancer cell clones acquire drug resistance; these cells gradually become predominant in the population, while other cancer cells that are sensitive to the drug die. To elucidate the mechanism of 5-FU resistance, we established three colon cancer cell lines which were resistant to 16 µM 5-FU administered continuously. Expression analysis of apoptosis-related proteins indicated a marked induction of anti-apoptotic BCL2 expression in the 5-FU-resistant HT-29 cells, consistent with previous studies that showed little to no expression of BCL2 in wild-type HT-29 colon cancer cells (16,17) and BCL2 stabilization in 5-FU-resistant HT-29 cells (18). However, our results showed that ABT-199, a BCL2 inhibitor, had no anti-apoptotic effect on 5-FU-resistant HT-29 cells, suggesting that BCL2 induction did not contribute to cell survival.

In general, a complex network comprising many apoptosis-related proteins makes it difficult to predict apoptotic responses merely by quantifying protein expression. Recently, a new functional assay, namely, BH3 profiling, was reported (15); this technique was shown to predict sensitivity to a single drug in patients with acute myelogenous leukemia (19), multiple myeloma (20) and gastric cancer (21). Based on our BH3 profiling results, 5-FU-resistant HT-29 colon cancer cells have specific BCLXL dependence, irrespective of the induction and expression of several apoptosis-related proteins. Disparate results between the quantification of protein expression and BH3 profiling provides great insight in clinical settings, as expression analysis including immunohistochemistry and real-time PCR has been conducted previously for assessing cancer cells. Therefore, to accurately assess protein dependence for cancer cell survival, BH3 profiling stands to become an auxiliary tool for targeted therapy, even after tumors acquire drug resistance.

BIM was induced in 5-FU-resistant HT-29 cells (Fig. 2A) and this protein enhanced sequestration by BCLXL (Fig. 6B). BIM, which is usually bound to microtubules under physiological conditions and recruited to mitochondria after treatment with cytotoxic drugs (22), functions as a pro-apoptotic activator of BAK and BAX (15). In our study, sequestration of induced BIM by BCLXL in 5-FU-resistant HT-29 cells resulted in the evasion of apoptosis caused by 5-FU; consequently, this BIM sequestration resulted in dependence on BCLXL even in the absence of 5-FU treatment, whereas parental HT-29 cells without BIM induction were unprimed for apoptosis to BCLXL-related BH3 peptide (Fig. 3A and D-F).

Similar to HT-29 cells, increased mitochondrial depolarization in 5-FU-resistant HCT-15 cells was also observed upon treatment with the XXA1 peptide (Fig. 3A). However, this BCLXL dependence is not associated with 5-FU resistance, as parental HCT-15 cells already tended to be primed by this XXA1 peptide. These results prompted us to consider trying a combination of 5-FU and BCLXL inhibitor prior to the acquisition of 5-FU resistance. However, as BCLXL plays a pivotal role in determining platelet life span (23), the combination of BCLXL inhibitor with a cytotoxic drug may cause severe thrombocytopenia. Indeed, clinical studies targeting BCLXL in glioblastoma multiforme (NCT00540722; http://clinicaltrials.gov/ct2/show/NCT00540722) and small cell lung cancer (NCT03080311; http://clinicaltrials.gov/ct2/show/NCT03080311) are ongoing; monotherapy has been conducted in these studies. Therefore, when treating cancers with a BH3 profiling pattern similar to parental and 5-FU-resistant HCT-15 cells, BCLXL-inhibitor treatment may be better after acquiring 5-FU resistance.

There are certain limitations to our study. Although BCLXL inhibition in 5-FU-resistant HT-29 cells may reverse sensitivity (Fig. 5B and C), the detailed mechanism by which BCLXL dependence affects cell survival exclusively in 5-FU-resistant HT-29 cells remains unknown. Furthermore, though BH3 profiling may distinguish a cell line sensitive to WEHI-539 out of three colon cancer cell lines *in vitro*, the efficacy of this profiling *in vivo* remains to be determined. Therefore, BH3 profiling of colon cancer samples before and after chemotherapy in clinical settings will be required.

In conclusion, our research involving BH3 profiling demonstrated a clear dependence on BCLXL in the acquisition of 5-FU drug resistance in HT-29 colon cancer cells among three colon cancer cell lines. In addition, we showed that the sequestration of the apoptosis-related BIM protein by BCLXL results in dependence on the latter protein. Clinical studies targeting BCLXL in laryngeal (NCT01633541; http://clinicaltrials.gov/ct2/show/NCT01633541) and small cell lung cancer (NCT03080311) are ongoing. Assessing BCLXL dependence in colon cancer cells through BH3 profiling will enable a more detailed stratification of individual sensitivities to this class of BCLXL selective inhibitors, thereby increasing the efficacy of precision medicine.

## Supplementary Material

Supporting Data

## Figures and Tables

**Table I.** Sequences of the BH3 peptide.

Name	Sequence
BIM	MRPEIWIAQELRRIGDEFNA
BID	EDIIRNIARHLAQVGDSMDRY
BAD	LWAAQRYGRELRRMSDEFEGSFKGL
NOXA	AELPPEFAAQLRKIGDKVYC
PUMA	EQWAREIGAQLRRMADDLNA
BMF	HQAEVQIARKLQLIADQFHRY
HRK	WSSAAQLTAARLKALGDELHQ
MS1	RPEIWMTQGLRRLGDEINAYYAR
XXA1	RPEIWYAQGLKRFGDEFNAYYAR

All BH3 peptides were 20–25 amino acid residues in length. The conserved LXXXXD motif was present in all peptides. All peptides were N-terminally acetylated and C-terminally amidated.

## Data Availability

The datasets used and/or analyzed during the current study are available from the corresponding author upon reasonable request.

## References

[1] Arnold M, Sierra MS, Laversanne M, Soerjomataram I, Jemal A, Bray F (2017). Global patterns and trends in colorectal cancer incidence and mortality. Gut.

[2] Hori M, Matsuda T, Shibata A, Katanoda K, Sobue T, Nishimoto H, Japan Cancer Surveillance Research Group (2015). Cancer incidence and incidence rates in Japan in 2009: A study of 32 population-based cancer registries for the Monitoring of Cancer Incidence in Japan (MCIJ) project. Jpn J Clin Oncol.

[3] Watanabe T, Muro K, Ajioka Y, Hashiguchi Y, Ito Y, Saito Y, Hamaguchi T, Ishida H, Ishiguro M, Ishihara S (2018). Japanese society for cancer of the colon and rectum (JSCCR) guidelines 2016 for the treatment of colorectal cancer. Int J Clin Oncol.

[4] Rutman RJ, Cantarow A, Paschkis KE (1954). Studies in 2-acetylaminofluorene carcinogenesis. III. The utilization of uracil-2-C14 by preneoplastic rat liver and rat hepatoma. Cancer Res.

[5] Longley DB, Harkin DP, Johnston PG (2003). 5-fluorouracil: Mechanisms of action and clinical strategies. Nat Rev Cancer.

[6] Liu W, Fang Y, Wang XT, Liu J, Dan X, Sun LL (2014). Overcoming 5-Fu resistance of colon cells through inhibition of Glut1 by the specific inhibitor WZB117. Asian Pac J Cancer Prev.

[7] Dallas NA, Xia L, Fan F, Gray MJ, Gaur P, van Buren G, Samuel S, Kim MP, Lim SJ, Ellis LM (2009). Chemoresistant colorectal cancer cells, the cancer stem cell phenotype, and increased sensitivity to insulin-like growth factor-I receptor inhibition. Cancer Res.

[8] Yoshida M, Horiguchi H, Kikuchi S, Iyama S, Ikeda H, Goto A, Kawano Y, Murase K, Takada K, Miyanishi K (2019). miR-7977 inhibits the Hippo-YAP signaling pathway in the bone marrow mesenchymal stromal cells. PLoS One.

[9] Ryan J, Letai A (2013). BH3 profiling in whole cells by fluorimeter or FACS. Methods.

[10] Fraser C, Ryan J, Sarosiek K (2019). BH3 profiling: A functional assay to measure apoptotic priming and dependencies. Methods Mol Biol.

[11] Tao ZF, Hasvold L, Wang L, Wang X, Petros AM, Park CH, Boghaert ER, Catron ND, Chen J, Colman PM (2014). Discovery of a potent and selective BCL-XL inhibitor with in vivo activity. ACS Med Chem Lett.

[12] Jian YS, Chen CW, Lin CA, Yu HP, Lin HY, Liao MY, Wu SH, Lin YF, Lai PS (2017). Hyaluronic acid-nimesulide conjugates as anticancer drugs against CD44-overexpressing HT-29 colorectal cancer in vitro and in vivo. Int J Nanomedicine.

[13] Dutta S, Ryan J, Chen TS, Kougentakis C, Letai A, Keating AE (2015). Potent and specific peptide inhibitors of human pro-survival protein Bcl-xL. J Mol Biol.

[14] Foight GW, Ryan JA, Gullá SV, Leta A, Keating AE (2014). Designed BH3 peptides with high affinity and specificity for targeting Mcl-1 in cells. ACS Chem Biol.

[15] Montero J, Letai A (2018). Why do BCL-2 inhibitors work and where should we use them in the clinic?. Cell Death Differ.

[16] Nita ME, Nagawa H, Tominaga O, Tsuno N, Fujii S, Sasaki S, Fu CG, Takenoue T, Tsuruo T, Muto T (1998). 5-Fluorouracil induces apoptosis in human colon cancer cell lines with modulation of Bcl-2 family proteins. Br J Cancer.

[17] Zhao DP, Ding XW, Peng JP, Zheng YX, Zhang SZ (2005). Prognostic significance of bcl-2 and p53 expression in colorectal carcinoma. J Zhejiang Univ Sci B.

[18] Wu DW, Huang CC, Chang SW, Chen TH, Lee H (2015). Bcl-2 stabilization by paxillin confers 5-fluorouracil resistance in colorectal cancer. Cell Death Differ.

[19] Vo TT, Ryan J, Carrasco R, Neuberg D, Rossi DJ, Stone RM, Deangelo DJ, Frattini MG, Letai A (2012). Relative mitochondrial priming of myeloblasts and normal HSCs determines chemotherapeutic success in AML. Cell.

[20] Ni Chonghaile T, Sarosiek KA, Vo TT, Ryan JA, Tammareddi A, Moore Vdel G, Deng J, Anderson KC, Richardson P, Tai YT (2011). Pretreatment mitochondrial priming correlates with clinical response to cytotoxic chemotherapy. Science.

[21] Kubo T, Kawano Y, Himuro N, Sugita S, Sato Y, Ishikawa K, Takada K, Murase K, Miyanishi K, Sato T (2016). BAK is a predictive and prognostic biomarker for the therapeutic effect of docetaxel treatment in patients with advanced gastric cancer. Gastric Cancer.

[22] Klotz DM, Nelson SA, Kroboth K, Newton IP, Radulescu S, Ridgway RA, Sansom OJ, Appleton PL, Näthke IS (2012). The microtubule poison vinorelbine kills cells independently of mitotic arrest and targets cells lacking the APC tumour suppressor more effectively. J Cell Sci.

[23] Yan Y, Xie R, Zhang Q, Zhu X, Han J, Xia R (2019). Bcl-xL/Bak interaction and regulation by miRNA let-7b in the intrinsic apoptotic pathway of stored platelets. Platelets.

