# Supplementary material for: BH3 profiling discriminates the anti-apoptotic status of 5-fluorouracil-resistant colon cancer cells
Source: Oncol Rep. 2019 Oct 15;42(6):2416–25. doi: 10.3892/or.2019.7373 (PMC6826312; doi:10.3892/or.2019.7373)

Figure S1. Apoptosis analysis by flow cytometry. Representative contour plot results of cells stained by Annexin V-PE and 7-AAD. Parental and 5-FU-resistant colon cancer cell lines were treated with 100 μM 5-fluorouracil (5-FU) for 72 h, followed by the assessment of apoptosis by flow cytometry.

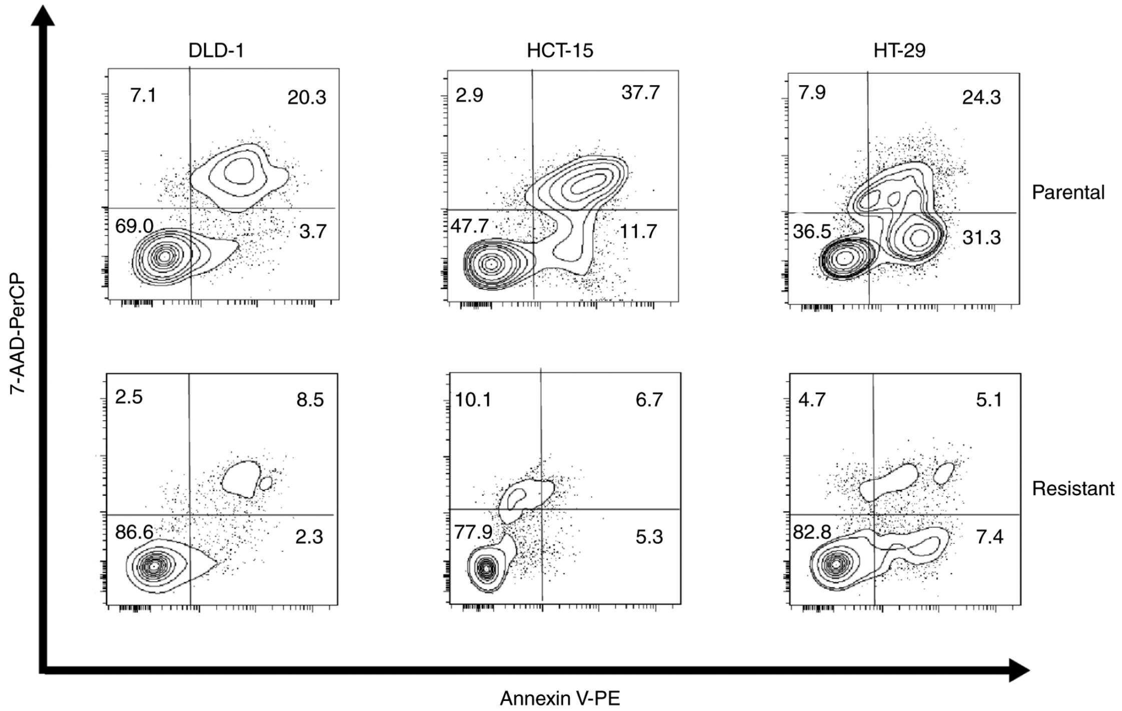

Figure S2. Images of mice with subcutaneous tumors at day 32 after treatment. There are 5 mice in each group.

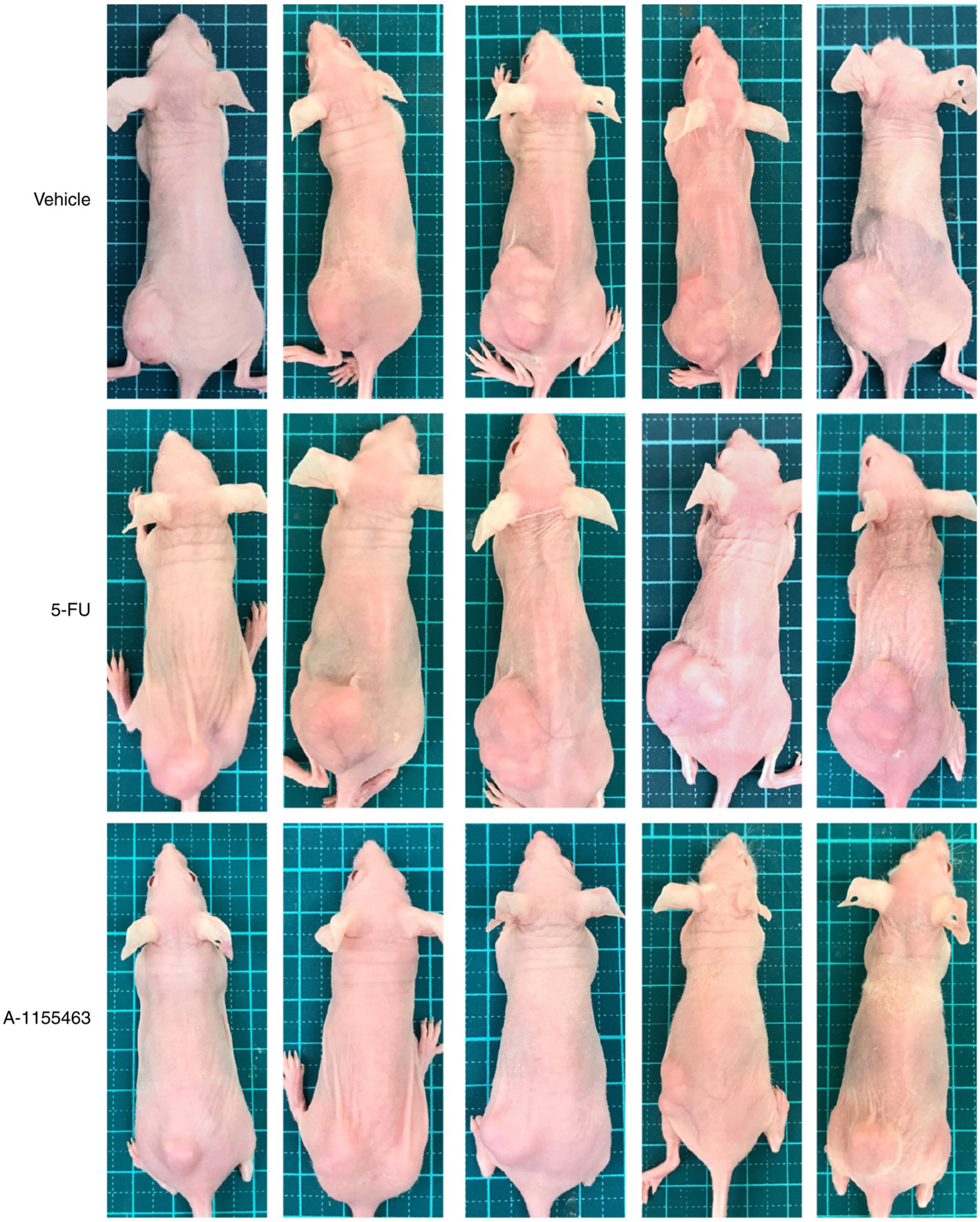

Figure S3. Body weight of mice treated with BCLXL inhibitor, A-1155463. Body weight of mice was monitored every 3 to 4 days. Data represent the mean ± SD (n=5).

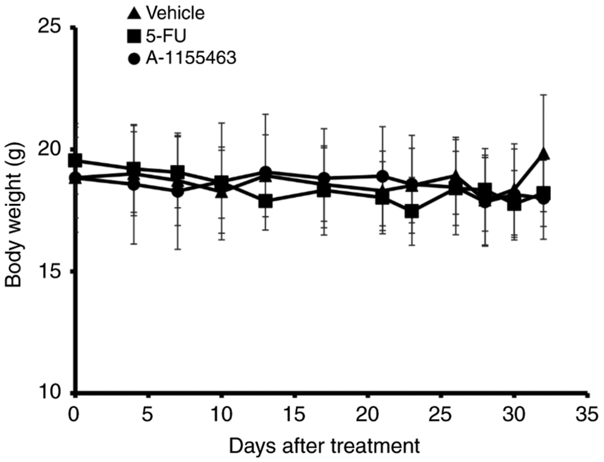

Figure S4. Histologic analysis of tumor sections stained with hematoxylin and eosin (H&E) and TUNEL. Scale bars, 100 μm.

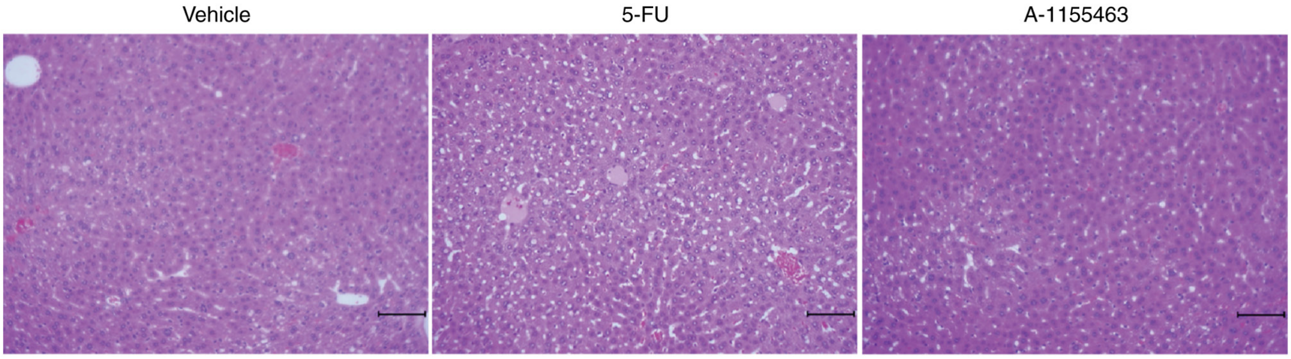

Supplement: Supporting Data [file Supplementary_Data.pdf]
